# Short and long-term outcomes of elderly patients undergoing left-sided colorectal resection with primary anastomosis for cancer

**DOI:** 10.1186/s12877-021-02648-2

**Published:** 2021-12-07

**Authors:** Marius Kryzauskas, Augustinas Bausys, Justas Kuliavas, Klaudija Bickaite, Audrius Dulskas, Eligijus Poskus, Rimantas Bausys, Kestutis Strupas, Tomas Poskus

**Affiliations:** 1 Clinic of Gastroenterology, Nephrourology, and Surgery, Institute of Clinical Medicine, Faculty of Medicine, Vilnius University, Vilnius, Lithuania; 2 Centre for Visceral Medicine and Translational Research, Institute of Clinical Medicine, Faculty of Medicine, Vilnius University, Vilnius, Lithuania; 3 Faculty of Medicine, Vilnius University, Vilnius, Lithuania

**Keywords:** Colorectal cancer, Elderly, Morbidity, Mortality, Anastomotic leakage

## Abstract

**Background:**

The proportion of elderly colorectal cancer (CRC) patients requiring surgery is increasing. Colorectal resection for left-sided cancers is the most controversial as the primary anastomosis or end-colostomy and open or minimally invasive approaches are available. Therefore, this study was conducted to investigate the short- and long-term outcomes in elderly patients after resection with primary anastomosis for left-sided CRC.

**Methods:**

The cohort study included left-sided colorectal cancer patients who underwent resection with primary anastomosis. The participants were divided into non-elderly (≤75 years) and elderly (> 75 years) groups. Short- and long-term postoperative outcomes were investigated.

**Results:**

In total 738 (82%) and 162 (18%) patients were allocated to non-elderly and elderly groups, respectively. Minimally invasive surgery (MIS) was less prevalent in the elderly (42.6% vs 52.7%, *p* = 0.024) and a higher proportion of these suffered severe or lethal complications (15.4% vs 9.8%, *p* = 0.040). MIS decreased the odds for postoperative complications (OR: 0.41; 95% CI: 0.19–0.89, *p* = 0.038). The rate of anastomotic leakage was similar (8.5% vs 11.7%, *p* = 0.201), although, in the case of leakage 21.1% of elderly patients died within 90-days after surgery. Overall- and disease-free survival was impaired in the elderly. MIS increased the odds for long-term survival.

**Conclusions:**

Elderly patients suffer more severe complications after resection with primary anastomosis for left-sided CRC. The risk of anastomotic leakage in the elderly and non-elderly is similar, although, leakages in the elderly seem to be associated with a higher 90-day mortality rate. Minimally invasive surgery is associated with decreased morbidity in the elderly.

## Introduction

Colorectal cancer (CRC) is a major health care issue as it is the third most deadly and fourth most commonly diagnosed cancer worldwide [1]. Surgery remains the only potentially curative treatment option for it [2]. As society is aging in many developed countries, the proportion of elderly patients requiring surgery for CRC is increasing as well [3, 4]. Despite improvements in perioperative care and surgical techniques, the treatment of elderly CRC patients remains challenging because of comorbidities, frailty, malnutrition, impaired functional, and cognitive status [5–8]. Such complex patients are at higher risk for various postoperative complications after major surgery, including a higher risk for infectious complications and anastomotic leakage (AL) [9–12]. Furthermore, elderly patients are at higher risk for death in case of postoperative complications because of the impaired functional reserve [13, 14]. These risks usually impact the surgeon’s decision on the surgical plan, especially for elderly patients with left-sided CRC where Hartmann’s procedure may be selected instead of primary anastomosis [15, 16]. Further, advanced age had initially been viewed as a relative contraindication to laparoscopic surgery [17], and minimally invasive surgery (MIS) is still underutilized in the elderly [18]. Since elderly patients are significantly underrepresented in the clinical studies due to careful participant selection by common age, performance status, or comorbidities restrictions [19, 20], there is a lack of evidence for the most appropriate surgical strategies in such patients. Therefore, this study was conducted to investigate the short- and long-term outcomes after resection with primary anastomosis for left-sided CRC in elderly patients, with a special focus on the rate of AL and utilization of MIS.

## Materials and methods

### Ethics

The study was approved by Vilnius Regional Bioethics Committee (Approval number 2019/3–116-608) and conducted according to the Declaration of Helsinki of 1964, as revised in later versions.

### Patients and study design

This retrospective cohort study included all patients who underwent elective colorectal resection with primary anastomosis at two major gastrointestinal cancer treatment centers in Lithuania – National Cancer Institute and Vilnius University hospital Santaros Klinikos between January 2014 and December 2018. Patients were divided into non-elderly (NE; ≤75 years) and elderly groups (E; > 75 years) according to the age at the time of surgery.

### Data collection

The database used for the present study was used previously [12]. All patients’ characteristics and clinical data were obtained from the medical records and prospectively collected databases. The preoperative data included: age, gender, body mass index (BMI), Charlson comorbidity index (CCI), comorbidities, American Society of Anesthesiology (ASA) score, type of neoadjuvant treatment, tumor localization. Chronic kidney failure was defined as a kidney damage or glomerular filtration rate (GFR) < 60 mL/min/1.73 m^2^ for 3 months or more, irrespective of cause as proposed by Kidney Disease: Improving Global Outcomes (KDIGO) [21]. Intraoperative details included: type of surgery, the approach of surgery (open or minimally invasive), operation time, blood loss, the height of anastomosis measured from the anal verge, presence of diverting ileostomy. Standard laparoscopic colorectal resection, hand-assisted laparoscopic surgery, natural orifice specimen extraction surgery, and transanal total mesorectal excision operations were defined as minimally invasive approaches. Postoperative data included histological report results, hospitalization time, postoperative complications graded by Clavien-Dindo classification, 30-day, and 90-day mortality rates. The tumor stage was set according to the TNM system as described at the American Joint Committee on Cancer 8th edition.

### Study outcomes

The primary outcome of the study was the anastomotic leakage rate in NE and E patients. The secondary outcomes were overall postoperative morbidity rate; in-hospital, 30-day, and 90-day mortality rates; the rate of MIS; overall survival (OS), and disease-free survival (DFS) rates in NE and E patients. OS was defined as the time from surgery to death. DFS was defined as the time from surgery to disease progression including local or distant recurrence or death. Data on survival and date of death were collected from the National Lithuanian Cancer registry.

### Statistical analysis

All statistical analyses were performed using SPSS version 25.0 software (SPSS, Chicago, IL, USA). Continuous variables between groups were compared by Student’s t-test or Mann– Whitney U-test depending on data distribution and expressed as mean ± standard deviation (±SD) or median with first (Q1) and third (Q3) quartiles. Categorical variables were compared by χ2 test or Fisher’s exact test and expressed as proportion and percentages. Missing data was not handled at the statistical analysis and no imputation techniques were used. To determine the risk factors for anastomotic leakage, all potential risk factors were included in univariate analyses. These variables which showed significance were included in subsequent multivariable analysis. Kaplan-Meier method was used for OS and DFS analysis and curves were compared by the log-rank test. Multivariable survival analysis was performed using the Cox proportional hazards model (hazard ratio and 95% confidence intervals). Statistical significance was assumed for *p* values < 0.05.

## Results

### Patients baseline characteristics

A total of 900 patients were included in this study. Seven hundred thirty-eight (82%) patients were allocated to the NE group (≤75 years) and 162 (18%) patients were allocated to the E group (> 75 years). Baseline clinical characteristics of the study patients are presented in Table 1. E patients had higher ASA and CCI scores, but a lower proportion of these was obese (Table 1).

**Table 1:** Baseline clinical characteristics of non-elderly and elderly patients

		NE group (≤75 years); *n* = 738	Missing data; n (%)	E group (> 75 years); *n* = 162	Missing data; n (%)	*p* value
BMI; n (%)	< 30	496 (71.2%)	41 (5.8%)	128 (84.2%)	10 (6.2%)	0.001
	≥30	201 (28.8%)		24 (15.8%)		
Gender; n (%)	Female	344 (46.6%)	0 (0%)	73 (45.1%)	0 (0%)	0.729
	Male	394 (53.4%)		89 (54.9%)		
ASA; n (%)	I-II	535 (76.0%)	34 (4.6%)	55 (35.7%)	8 (4.9%)	0.001
	III-IV	169 (24.0%)		99 (64.3%)		
CCI; n (%)	≤5	603 (81.7%)	0 (0%)	47 (29.0%)	0 (0%)	0.001
	> 5	135 (18.3%)		115 (71.0%)		
Ischemic heart disease; n (%)	Yes	26 (3.5%)	0 (0%)	17 (10.5%)	0 (0%)	0.001
	No	712 (96.5%)		145 (89.5%)		
Diabetes mellitus; n (%)	Yes	71 (9.6%)	0 (0%)	24 (14.8%)	0 (0%)	0.065
	No	667 (90.4%)		138 (85.2%)		
Cerebrovascular disease; n (%)	Yes	18 (2.4%)	0 (0%)	9 (5.6%)	0 (0%)	0.043
	No	720 (97.6%)		153 (94.4%)		
Chronic kidney failure; n (%)	Yes	9 (1.2%)	0 (0%)	4 (2.5%)	0 (0%)	0.267
	No	729 (98.8%)		158 (97.5%)		
Neoadjuvant treatment; n (%)	Yes	163 (22.1%)	0 (0%)	29 (17.9%)	0 (0%)	0.289
	No	575 (77.9%)		133 (82.1%)		
Specimen length, cm (Mean ± SD)		19 ± 8	22 (2.9%)	21 ± 6	4 (2.4%)	0.436
Proximal end, cm (Mean ± SD)		13 ± 7	27 (3.6%)	13 ± 6	5 (3.0%)	0.346
Distal end, cm (Mean ± SD)		4 ± 3	26 (3.5%)	4 ± 4	5 (3.0%)	0.109
T; n (%)	T0–2	271 (36.7%)	0 (0%)	30 (18.5%)	0 (0%)	0.001
	T3–4	467 (63.3%)		132 (81.5%)		
N; n (%)	N0	449 (61.8%)	12 (1.6%)	92 (57.1%)	1 (0.6%)	0.284
	N+	277 (38.2%)		69 (42.9%)		
M; n (%)	M0	666 (90.2%)	0 (0%)	144 (88.9%)	0 (0%)	0.566
	M1	72 (9.8%)		18 (11.1%)		
Stage; n (%)	0	11 (1.5%)	0 (0%)	1 (0.6%)	0 (0%)	0.002
	I	203 (27.5%)		23 (14.2%)		
	II	205 (27.8%)		64 (39.5%)		
	III	246 (33.3%)		56 (34.6%)		
	IV	73 (9.9%)		18 (1.1%)		

### Intraoperative and postoperative outcomes

Intraoperative and postoperative outcomes are shown in Table 2. Lower proportion of E patients received MIS (52.7% vs 42.6%, *p* = 0.024). There was some tendency for a higher postoperative morbidity rate in the E (37.0%) group compared to NE (29.7%) group, however, the difference failed for significance (*p* = 0.066). Although, severe or lethal complications by Clavien-Dindo score III-V were more common in the E group (15.4% vs 9.8%, *p* = 0.040).

**Table 2:** Intraoperative and postoperative outcomes of non-elderly and elderly patients after resection with primary anastomosis for left-sided colorectal cancer

		NE group (≤75 years); *n* = 738	E group (> 75 years); *n* = 162	*p* value
Type of surgery; n (%)	Sigmoid resection	214 (29.0%)	43 (26.5%)	0.565
	Rectal resection	524 (71.0%)	119 (73.5%)	
Approach of surgery; n (%)	Open	349 (47.3%)	93 (57.4%)	0.024
	Minimally invasive	389 (52.7%)	69 (42.6%)	
Operation time, minutes (mean ± SD)		147 ± 60	150 ± 67	0.190
Blood loss, ml (median; Q1, Q3)		50 (Q1: 50; Q3: 100)	100 (Q1: 50; Q3: 162)	0.522
Anastomosis level from anal verge; n (%)	≤5 cm	145 (23.7%)	29 (22.3%)	0.860
	6–12 cm	239 (39.0%)	54 (41.5%)	
	> 12 cm	229 (37.3%)	47 (36.2%)	
Diverting ileostomy; n (%)	Yes	302 (40.9%)	72 (44.4%)	0.429
	No	436 (59.1%)	90 (55.6%)	
Postoperative hospitalization; days (mean ± SD)		10 ± 6	13 ± 11	0.001
Retrieved lymph nodes; n (%)	< 12	130 (17.6%)	18 (11.1%)	0.046
	≥12	608 (82.4%)	144 (88.9%)	
Postoperative complications; n (%)	Yes	219 (29.7%)	60 (37.0%)	0.066
	No	519 (70.3%)	102 (63.0%)	
Severe complications by Clavien-Dindo score III-V; n (%)		73 (9.8%)	25 (15.4%)	0.040
30-day mortality; n (%)		7 (0.9%)	5 (3.1%)	0.048
90-day mortality; n (%)		12 (1.6%)	12 (7.4%)	0.001

### Anastomotic leakage in the study cohort

Eighty-two of 900 (9.1%) patients included in the study developed AL. Male gender, higher CCI score (> 5), advanced pT stage (pT3–4), lower anastomoses, and open surgery were associated with AL in the univariate analysis (Table 3). The rate of AL was similar between NE (8.5%) and E (11.7%) groups, *p* = 0.201. Although, there was some tendency for increased 90-days mortality in E patients who developed AL, but without statistical significance (6.3% vs 21.1%, *p* = 0.079). Variables that showed significance in univariate analysis were included in subsequent multivariable analysis. Male gender (OR: 1.94; 95% CI: 1.15–3.29, *p* = 0.013), CCI score > 5 (OR: 1.90; 95% CI: 1.14–3.16, p = 0.013), and anastomoses at 6–12 cm from anal verge (OR: 2.29; 95% CI: 1.24–4.21, *p* = 0.008) were identified as a risk factor for AL (Table 4).

**Table 3:** Univariate analysis of risk factors for anastomotic leakage in patients after resection with primary anastomosis for left-sided colorectal cancer

		No anastomotic leakage	Anastomotic leakage	*p* value
Gender; n (%)	Female	391 (93.8%)	26 (6.2%)	0.005
	Male	427 (88.4%)	56 (11.6%)	
CCI; n (%)	≤5	602 (92.6%)	48 (7.4%)	0.004
	> 5	216 (86.4%)	34 (13.6%)	
Ischemic heart disease; n (%)	Yes	39 (90.7%)	4 (9.3%)	0.999
	No	779 (90.9%)	78 (9.1%)	
Diabetes mellitus; n (%)	Yes	82 (86.3%)	13 (13.7%)	0.101
	No	736 (91.4%)	69 (8.6%)	
Cerebrovascular disease; n (%)	Yes	25 (92.6%)	2 (7.4%)	0.999
	No	793 (90.8%)	80 (9.2%)	
Chronic kidney failure; n (%)	Yes	12 (92.3%)	1 (7.7%)	0.999
	No	806 (90.9%)	81 (9.1%)	
Neoadjuvant treatment; n (%)	Yes	169 (88.0%)	23 (12.0%)	0.119
	No	649 (91.7%)	59 (8.3%)	
Tumor localization; n (%)	Rectum	458 (89.3%)	55 (10.7%)	0.132
	Rectosigmoid	112 (91.8%)	10 (8.2%)	
	Sigmoid	248 (93.6%)	17 (6.4%)	
T; n (%)	T0–2	284 (94.4%)	17 (5.6%)	0.010
	T3–4	534 (89.1%)	65 (10.9%)	
M; n (%)	M0	740 (91.4%)	70 (8.6%)	0.142
	M1	78 (86.7%)	12 (13.3%)	
Stage; n (%)	0	11 (91.7%)	1 (8.3%)	0.290
	I	221 (93.4%)	15 (6.6%)	
	II	246 (91.4%)	23 (8.6%)	
	III	272 (90.1%)	30 (9.9%)	
	IV	78 (85.7%)	13 (14.3%)	
Ligation of inferior mesenteric artery; n (%)	High	631 (90.5%)	66 (9.5%)	0.610
	Low	167 (91.8%)	15 (8.2%)	
Simultaneous operation; n (%)	Yes	73 (86.9%)	11 (13.1%)	0.183
	No	745 (91.3%)	71 (8.7%)	
Anastomosis level from anal verge; n (%)	≤5 cm	155 (89.1%)	19 (10.9%)	0.023
	6–12 cm	255 (87.0%)	38 (13.0%)	
	> 12 cm	259 (93.8%)	17 (6.2%)	
Approach of surgery; n (%)	Open	391 (88.5%)	51 (11.5%)	0.013
	Minimally invasive	427 (93.2%)	31 (6.8%)	
Age; n (%)	NE group (≤75 years)	675 (91.5%)	63 (8.5%)	0.201
	E group (>75 years)	143 (88.3%)	19 (11.7%)

**Table 4:** Multivariable analysis of risk factors for anastomotic leakage in patients after resection with primary anastomosis for left-sided colorectal cancer

Risk factor		Odds ratio (95% CI)	*p* value
Gender	Female	1 (Reference)	
	Male	1.94 (1.15–3.29)	0.013
CCI	≤5	1 (Reference)	
	> 5	1.90 (1.14–3.16)	0.013
pT stage	T0–2	1 (Reference)	
	T3–4	1.82 (0.97–3.42)	0.060
Anastomosis level from anal verge	> 12 cm	1 (Reference)	
	6–12 cm	2.29 (1.24–4.21)	0.008
	≤5 cm	1.90 (0.93–3.87)	0.076
Approach of surgery	Open	1 (Reference)	
	Minimally invasive	0.65 (0.39–1.09)	0.109

### Factors associated with postoperative morbidity in the subgroup of elderly patients

Since the E patients were at higher risk for postoperative morbidity and mortality, the univariate analysis was performed to identify the variables associated with postoperative complications in the subgroup of E patients. Open surgery was the only risk factor associated with postoperative complications in the univariate setting (Table 5).

**Table 5:** Univariate analysis of risk factors for postoperative complications in elderly patients after resection with primary anastomosis for left-sided colorectal cancer

		No postoperative complications	Postoperative complications	*p* value
Gender; n (%)	Female	51 (50.0%)	22 (36.7%)	0.106
	Male	51 (50.0%)	38 (63.3%)	
ASA; n (%)	I-II	40 (40.8%)	15 (26.8%)	0.115
	III-IV	58 (59.2%)	41 (73.2%)	
CCI; n (%)	≤5	33 (32.4%)	14 (23.3%)	0.283
	> 5	69 (67.6%)	46 (76.7%)	
Ischemic heart disease; n (%)	Yes	12 (11.8%)	5 (8.3%)	0.601
	No	90 (88.2%)	55 (91.7%)	
Diabetes mellitus; n (%)	Yes	16 (15.7%)	8 (13.3%)	0.820
	No	86 (84.3%)	52 (86.7%)	
Cerebrovascular disease; n (%)	Yes	7 (6.9%)	2 (3.3%)	0.487
	No	95 (93.1%)	58 (96.7%)	
Chronic kidney failure; n (%)	Yes	3 (2.9%)	1 (1.7%)	0.999
	No	99 (97.1%)	59 (98.3%)	
Neoadjuvant treatment; n (%)	Yes	19 (18.6%)	10 (16.7%)	0.834
	No	83 (81.4%)	50 (83.3%)	
Tumor localization; n (%)	Rectum	55 (53.9%)	33 (55.0%)	0.170
	Rectosigmoid	14 (13.7%)	14 (23.3%)	
	Sigmoid	33 (32.4%)	13 (21.7%)	
T; n (%)	T0–2	20 (19.6%)	10 (16.7%)	0.681
	T3–4	82 (80.4%)	50 (83.3%)	
M; n (%)	M0	92 (90.2%)	52 (86.7%)	0.606
	M1	10 (9.8%)	8 (13.3%)	
Stage; n (%)	0	1 (1.0%)	0 (0.0%)	0.735
	I	13 (12.7%)	10 (16.7%)	
	II	40 (39.2%)	24 (40.0%)	
	III	38 (37.3%)	18 (30.0%)	
	IV	10 (9.8%)	8 (13.3%)	
Ligation of inferior mesenteric artery; n (%)	High	71 (70.3%)	42 (72.4%)	0.857
	Low	30 (29.7%)	16 (27.6%)	
Simultaneous operation; n (%)	Yes	6 (5.9%)	8 (13.3%)	0.146
	No	96 (94.1%)	52 (86.7%)	
Anastomosis level from anal verge; n (%)	≤5 cm	16 (19.8%)	13 (26.5%)	0.351
	6–12 cm	32 (39.5%)	22 (44.9%)	
	> 12 cm	33 (40.7%)	14 (28.6%)	
Approach of surgery; n (%)	Open	52 (51.0%)	41 (68.3%)	0.034
	Minimally invasive	50 (49.0%)	19 (31.7%)	

### Survival

**Fig. 1:**
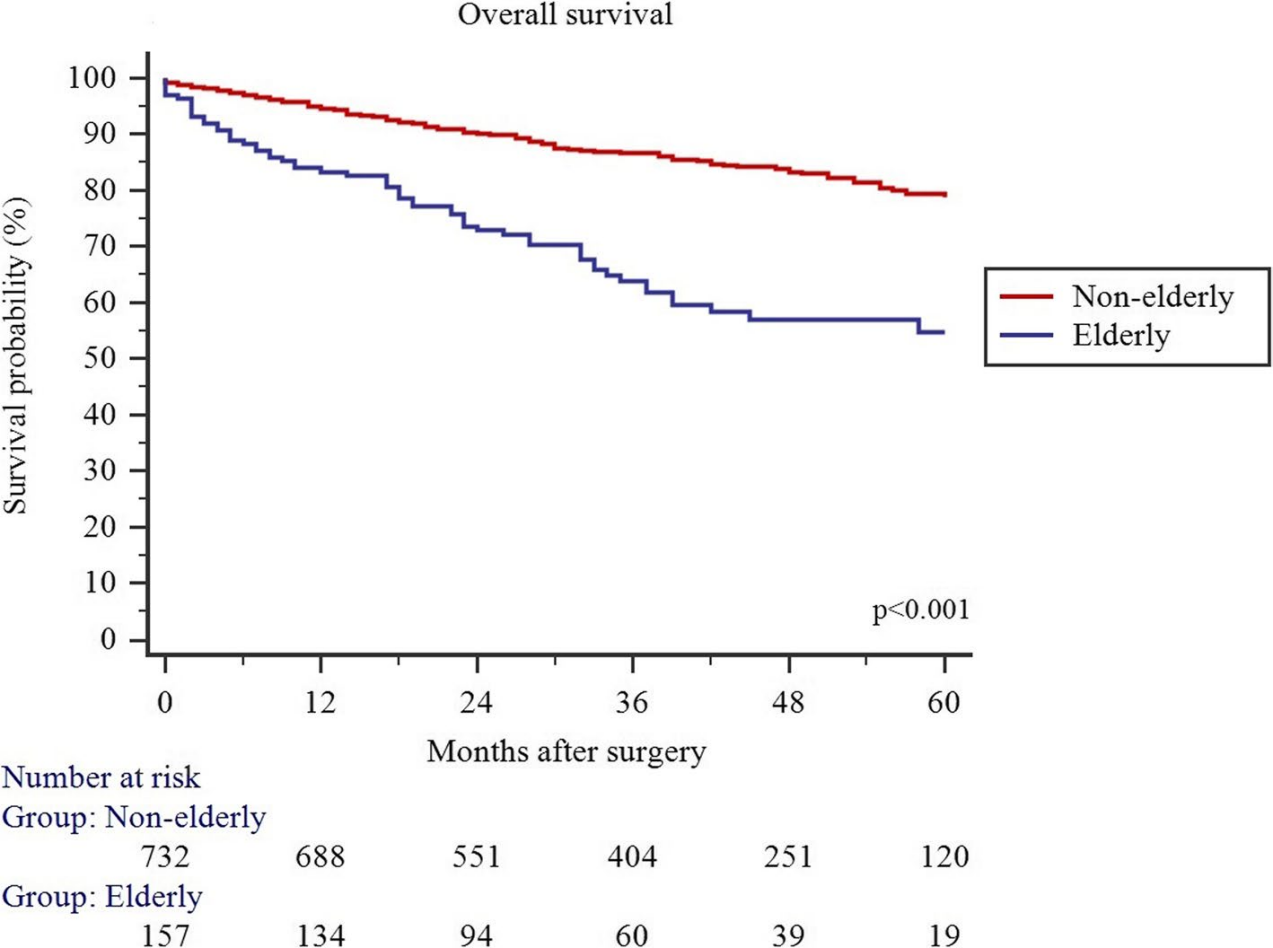
Overall survival in non-elderly and elderly patients who received colorectal resection with primary anastomosis for left-sided colorectal cancer

**Fig. 2:**
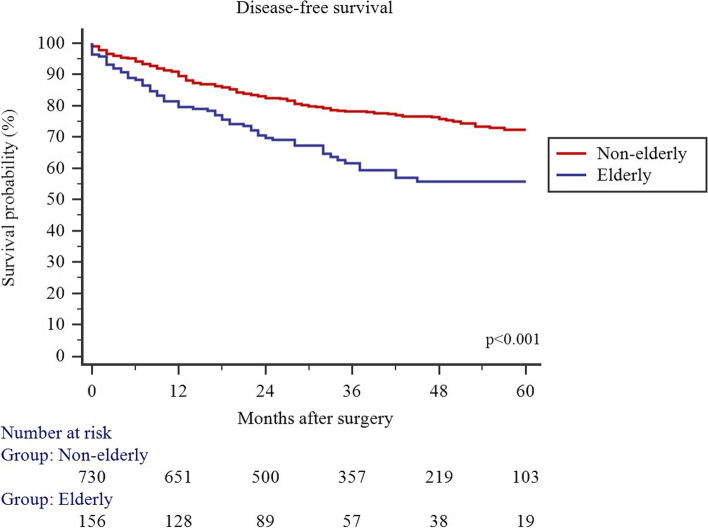
Disease-free survival in non-elderly and elderly patients who received colorectal resection with primary anastomosis for left-sided colorectal cancer

The median time to follow-up was 38 (Q1: 22; Q3: 53) months. Overall and disease-free survival was significantly lower in E patients (Figs. 1 and 2). The multivariable Cox proportional hazards model was performed to identify the factors associated with OS and DFS in the E group. E patients who received MIS had higher probability for OS (HR: 0.47; 95% CI: (0.25–0.86), *p* = 0.015) and DFS (HR: 0.48; 95% CI: (0.27–0.86) (Table 6).

**Table 6:** Cox regression (multivariable) analysis for overall and disease-free survival in the elderly patients after resection with primary anastomosis for left-sided CRC

		Overall survival		Disease-free survival	
		HR (95% CI)	*p*	HR (95% CI)	*p*
Gender	Female	1 (reference)		1 (reference)	
	Male	1.20 (0.68–2.13)	0.521	1.18 (0.67–2.07)	0.558
pT	T0–2	1 (reference)		1 (reference)	
	T3–4	0.89 (0.41–1.93)	0.783	0.87 (0.41–1.85)	0.723
pN	N0	1 (reference)		1 (reference)	
	N+	1.28 (0.69–2.36)	0.422	1.46 (0.80–2.66)	0.210
pM	M0	1 (reference)		1 (reference)	
	M1	2.00 (0.69–2.36)	0.422	1.94 (0.82–4.57)	0.128
ASA score	I-II	1 (reference)		1 (reference)	
	III-IV	1.91 (0.98–3.75)	0.057	2.12 (1.09–4.12)	0.026
Postoperative complications	No	1 (reference)		1 (reference)	
	Yes	0.82 (0.41–1.63)	0.580	0.92 (0.47–1.80)	0.816
Anastomotic leakage	No	1 (reference)		1 (reference)	
	Yes	1.69 (0.68–4.19)	0.256	1.85 (0.78–4.38)	0.160
Surgical approach	Open	1 (reference)		1 (reference)	
	Minimally invasive	0.47 (0.25–0.86)	0.015	0.48 (0.27–0.86)	0.015
LN retrieval	≥12	1 (reference)		1 (reference)	
	< 12	0.52 (0.26–1.02)	0.060	0.53 (0.27–1.02)	0.058
Tumor localization	Sigmoid	1 (reference)		1 (reference)	
	Rectum	1.64 (0.71–3.81)	0.242	1.45 (0.63–3.35)	0.375

## Discussion

The present study demonstrated the trend of a slightly higher rate of postoperative morbidity in the elderly patients after colorectal resection with the primary anastomosis for left-sided cancer. However, the rate of severe or lethal complications was undoubtedly higher in the elderly patients group. Interestingly, the rate of AL was similar across the study groups, but in the case of leakage elderly patients were at much higher risk for death within 90-days after surgery. The MIS was associated with reduced postoperative morbidity in the elderly; however, this approach was underutilized in these patients.

The reported rate of postoperative complications in elderly colorectal cancer patients varies between 26 and 53.7% [22–24], as our study showed a comparable rate of 37%. The elderly patients often have a higher ASA score [25–27], which is the risk factor for postoperative complications as shown in the present study and some previous reports [22]. It is not surprising, that the frequent presence of comorbidities, frailty and impaired functional reserves in the elderly leads to the increased postoperative morbidity and mortality [23, 28–30]. However, it remains unclear if elderly patients are at a higher risk for all types of complications or only specific ones. The particularly important question is whether the elderly patients are at higher risk for the AL, especially after resection for left-sided cancer. This has special importance, because, the higher rate of AL compared to right-side surgery [31] is preventable by utilizing Hartmann’s procedure. The current data on the risk of AL in elderly patients is inconclusive. Some studies suggest a higher risk because of co-existing medical conditions, which are known risk factors for AL, such as coronary heart disease and diabetes [27, 32, 33]. In contrast, the other series of previous studies identified a similar risk of AL in elderly and non-elderly patients [34–37]. The present study shows that the risk in elderly and non-elderly patients after resection for left-sided CRC is similar. However, it is necessary to note, that the consequences of leakage in the elderly were much more dramatic since the 90-day mortality rate exceeded 20%. Thus, we consider, that primary anastomosis after left-sided resection for CRC is feasible in the elderly, but these patients must be monitored closely, and in the case of AL the aggressive treatment of the complication is mandatory.

MIS is currently considered an excellent alternative for open CRC surgery since large-scale RCTs demonstrated improved short-term and similar long-term outcomes [38–43]. Furthermore, large-scale population-based studies show that MIS is associated with decreased morbidity and mortality in CRC patients [44, 45]. Despite such evidence, MIS is underutilized in elderly patients as demonstrated by this study. A similar pattern of slow and even decreasing adoption of laparoscopic CRC surgery in the elderly is observed not only in our cohort but in other Western countries as well [18]. The reasons for such disparities in implementing MIS for younger and elderly CRC patients remain unclear. Although, some controversies exist on this topic and they may be responsible for the reluctance to perform MIS in the elderly. First, MIS is associated with significantly longer operative time, therefore there is a long time of the patient under anesthesia. Second, the potential cardiopulmonary changes induced by pneumoperitoneum and prolonged patient positioning remains a concern. Third, the studies which proved the benefit of MIS in CRC patients underrepresented the elderly population. Thus, there is a background for some scepticism regarding MIS adoption in elderly. Although, several previous studies showed the favourable outcomes of MIS in elderly CRC patients [46–50]. Further, our study confirmed, that MIS is associated with lower odds for postoperative complications in elderly patients who undergo resection with primary anastomosis for left-sided cancer. Hence, surgeons should not avoid MIS in the elderly, because this high-risk population seems to receive a significant benefit from this technique.

In contrast to some previous reports [51, 52], we found impaired long-term outcomes in elderly patients after resection for left-sided CRC. The first 3 months after surgery were suggested as the most critical for these patients [51] and the results of the present confirmed the importance of the early postoperative period as 90-days mortality reached 7.4% in elderly and only 1.6% in younger counterparts. Such findings indicate the need for remarkably close monitoring of late postoperative complications and life-threatening events during the early postoperative period in elderly population undergoing colorectal resection. To our surprise, we found impaired DFS in elderly patients as well. There is no clear explanation for such a finding since there is no evidence for a more aggressive biological behaviour of CRC in the elderly. However, few patients and treatment-related may be responsible. At first, the most frail elderly patients do not receive adjuvant chemotherapy because of poor physical condition [53]. Second, elderly patients are at higher risk for postoperative complications, which are responsible for the delay of adjuvant chemotherapy [54], thus the impaired oncological outcomes [55]. Third, elderly patients, who receive adjuvant therapy, are at higher risk for dose de-escalation because of renal and liver dysfunctions [3]. For these reasons, successful surgical treatment with an uneventful postoperative course plays a key role in the management of CRC in this population. As the present study showed, the MIS is an excellent option for elderly patients since the lower odds for postoperative morbidity, recurrence of disease, and death.

Our study has several limitations. First, it is a retrospective cohort study, therefore it is subject to the biases and confounding factors linked to such methods of research. Moreover, missing data was not handled at the statistical analysis and no imputation techniques were used as missing rate of < 5% is considered inconsequential. Second, there was an unidentifiable bias in the decisions to perform open or MIS in elderly patients. It is possible that the choice was made in settings of surgeon experience and the patient’s global health status, thus, lower morbidity after MIS may be the consequence of the selection bias, rather than the real advantage of the method. Third, this study did not include any patient-reported outcomes, such as quality of life or others.

A strength of the current multi-center study includes a large sample size of the left-sided CRC patients who receive resection with primary anastomosis with long-term survival data.

## Conclusions

Short- and long-term outcomes of elderly patients who underwent resections with primary anastomosis for left-sided CRC are impaired. The risk of anastomotic leakage in the elderly and non-elderly patients is similar, but leakages in the elderly seem to be associated with a higher 90-day mortality rate. Minimally invasive surgery is associated with decreased morbidity in the elderly and better long-term outcomes.

## Data Availability

The datasets used and/or analyzed during the current study are available from the corresponding author on reasonable request.

## Abbreviations

CRC: Colorectal cancer
AL: Anastomotic leakage
MIS: Minimally invasive surgery
NE: Non-elderly
E: Elderly
BMI: Body mass index
CCI: Charlson comorbidity index
ASA: American Society of Anesthesiology
OS: Overall survival
DFS: Disease-free survival
